# Effects of Exercise Modalities on Arterial Stiffness and Wave Reflection: A Systematic Review and Meta-Analysis of Randomized Controlled Trials

**DOI:** 10.1371/journal.pone.0110034

**Published:** 2014-10-15

**Authors:** Ammar W. Ashor, Jose Lara, Mario Siervo, Carlos Celis-Morales, John C. Mathers

**Affiliations:** 1 Human Nutrition Research Centre, Institute of Cellular Medicine, Newcastle University, Campus for Ageing and Vitality, Newcastle on Tyne, United Kingdom; 2 College of Medicine, University of Al-Mustansiriyah, Baghdad, Iraq; Shanghai Institute of Hypertension, China

## Abstract

**Background and Objectives:**

Physical activity is associated with lower cardiovascular and all-cause mortality. However, the effects of different exercise modalities on arterial stiffness are currently unclear. Our objectives were to investigate the effects of exercise modalities (aerobic, resistance or combined) on pulse wave velocity (PWV) and augmentation index (AIx), and to determine whether the effects on these indices differed according to the participants' or exercise characteristics.

**Methods:**

We searched the Medline, Embase and Cochrane Library databases from inception until April 2014 for randomized controlled trials lasting ≥4 weeks investigating the effects of exercise modalities on PWV and AIx in adults aged ≥18 years.

**Results:**

Forty-two studies (1627 participants) were included in this analysis. Aerobic exercise improved both PWV (WMD: −0.63 m/s, 95% CI: −0.90, −0.35) and AIx (WMD:−2.63%, 95% CI: −5.25 to −0.02) significantly. Aerobic exercise training showed significantly greater reduction in brachial-ankle (WMD: −1.01 m/s, 95% CI: −1.57, −0.44) than in carotid-femoral (WMD: -0.39 m/s, 95% CI: −0.52, −0.27) PWV. Higher aerobic exercise intensity was associated with larger reductions in AIx (β: −1.55%, CI −3.09, 0.0001). In addition, aerobic exercise had a significantly larger effect in reducing PWV (WMD:−1.0 m/s, 95% CI: −1.43, −0.57) in participants with stiffer arteries (PWV ≥8 m/s). Resistance exercise had no effect on PWV and AIx. There was no significant effect of combined exercise on PWV and AIx.

**Conclusions:**

We conclude that aerobic exercise improved arterial stiffness significantly and that the effect was enhanced with higher aerobic exercise intensity and in participants with greater arterial stiffness at baseline.

**Trial Registration PROSPERO:**

Database registration: CRD42014009744,.

## Introduction

Physical activity (PA) are associated with 35% reduction in cardiovascular disease (CVD) mortality and 33% reduction in all-cause mortality in comparison with sedentary lifestyle [1]. Moreover, there is an inverse dose-response relationship between PA and CVD mortality or risk of coronary artery diseases [2]. Changes in known CVD risk factors such as body weight, blood pressure and serum lipids explain a large proportion (59%) of the observed beneficial effect of exercise on major CVD outcomes [3]. The remaining 40% of risk reduction may be attributed to effects on vascular hemodynamics including endothelial function (EF), arterial remodelling and compliance [4].

Arterial stiffening is a hallmark of ageing and is closely associated with many pathological conditions including atherosclerosis, dyslipidaemia, diabetes and chronic kidney diseases [5]. Reduced arterial compliance (i.e. increased stiffness) leads to faster reflection of the systolic wave from the peripheral small arteries to the heart, causing augmentation of the central aortic pressure [6]. This augmentation in central pressure leads to increased ventricular afterload and reduced coronary perfusion pressure which, eventually, may cause myocardial hypertrophy, ischaemia and infarction [7]. Thus, arterial stiffness appears to contribute to the complex etiology of CVD and is regarded as a predictor of increased CVD risk and all-cause mortality [8].

Evidence from randomized controlled trials (RCT) demonstrated a beneficial effect of various exercise modalities (aerobic, resistance and combined) on endothelial function [9]–[11] but there is controversy about the effects of different exercise modalities on indices of arterial stiffness and wave reflection [12]–[14].

This systematic review aimed to investigate the effects of various exercise modalities on both PWV and AIx. The secondary aims were to determine whether the effects of exercise training on PWV and AIx were modified by participants (age, sex, health status, body mass index (BMI), heart rate, blood pressure, or baseline values of PWV) or exercise characteristics (intensity, frequency, duration of sessions or duration of intervention).

## Methods

This systematic review was conducted according to the Cochrane guidelines and is reported according to PRISMA guidelines [15], [16].

### Data sources

The search for relevant studies was conducted via electronic searches of three databases (MEDLINE, Embase, and Cochrane library from inception until April 2014). Additionally, we searched for eligible studies in the reference lists of the relevant articles and reviews. The following keywords were used to search the above databases: exercise, training, physical activity, arterial stiffness, pulse wave velocity, augmentation index.

### Study selection

The inclusion criteria for eligible studies were as follows: 1) RCT of exercise with comparative non-exercise, usual care or sedentary groups; 2) prescribed structured exercise intervention of ≥4 weeks duration; 3) adult humans aged ≥18 years; 4) studies that measured arterial stiffness by PWV or AIx before and after intervention. The selection of eligible studies was conducted by two reviewers independently (AA, JL). If consensus was reached, articles were excluded or moved to the next stage (full-text screening) as appropriate. If consensus was not reached the article was moved to the next stage where the full-text of selected articles was appraised. Disagreements were resolved by discussion between the reviewers until consensus was reached.

### Data extraction and quality assessment

The following information was extracted from eligible articles: 1) study design, quality, and sample size; 2) participants' characteristics (age, sex, health status, BMI, and baseline arterial stiffness and wave reflection indices, heart rate, systolic and diastolic blood pressure); 3) characteristics of exercise intervention (type, duration and frequency of sessions, and intensity and duration of intervention); 4) outcome measures and instruments; and 5) indices of study quality which was assessed by the modified Jadad's score (range 0–5) using three main items related to randomization, blinding and description of dropout or withdrawals [17]. Because it is difficult (if not impossible) to blind participants to an exercise intervention, we considered the blinding of the outcome assessment by the operator as a quality criterion.

### Statistical analyses

The outcome of the meta-analysis is the net difference between the intervention and control group in PWV or AIx at the end of the study. PWV is defined as the speed of travel of the pressure pulse along the arterial segment, is calculated as the distance/transit time ratio and is expressed as meters per second [18]. The AIx is defined as the proportion of central pulse pressure due to the late systolic peak which, in turn, is attributed to the reflected pulse wave and is expressed as percentage [19]. Statistical analyses were performed using STATA 12 (StataCorp. 2011. College Station, TX, USA). The effect size was estimated as weighted mean differences (WMDs) with 95% confidence interval. Random effect models were used to take account of between-study heterogeneity for participant characteristics, study design and methods used to assess arterial stiffness and wave reflection. Data not provided in the main text or tables were extracted from the figures. For crossover trials, we used the mean and SD separately for the intervention and control conditions [20]. In trials with multiple treatment arms and a single control group, the sample size of the control group was divided by the number of treatment groups to avoid over-inflation of the sample size [15].

Subgroup analyses were undertaken to investigate the role of potential factors influencing the effect of exercise modalities on arterial stiffness and accounting for heterogeneity in the models. These factors included the age, gender and health status of participants, baseline values for BMI, blood pressure, PWV and AIx. However, in this investigation we restricted subgroup analysis to cases with ≥5 studies per subgroup [15]. Meta-regression analyses were conducted to examine the relationships between exercise characteristics (intensity, frequency and duration of sessions) and changes in PWV and AIx indices. Where studies reported a range of intensities across different time periods, exercise intensity was calculated as the mean intensity across all periods. Both relative (% maximum heart rate, %HRmax) and absolute (metabolic equivalents, METs) exercise intensities were calculated using the methods and formulae described by Howley [21]. Where studies used both aerobic and resistance exercise interventions, we estimated the exercise intensities for each type separately and then calculated the average of both exercise intensities.

Publication bias was evaluated by visual inspection of the funnel plot and by Egger's regression test [22]. Heterogeneity between studies was evaluated using Cochrane Q statistics; *P*>0.1 indicates significant heterogeneity. The *I^2^* test was also used to evaluate consistency between studies where a value <25% indicates low risk of heterogeneity, 25–75% indicates moderate risk of heterogeneity and >75% indicates high risk of heterogeneity [23].

## Results

### Search results

**Figure 1:**
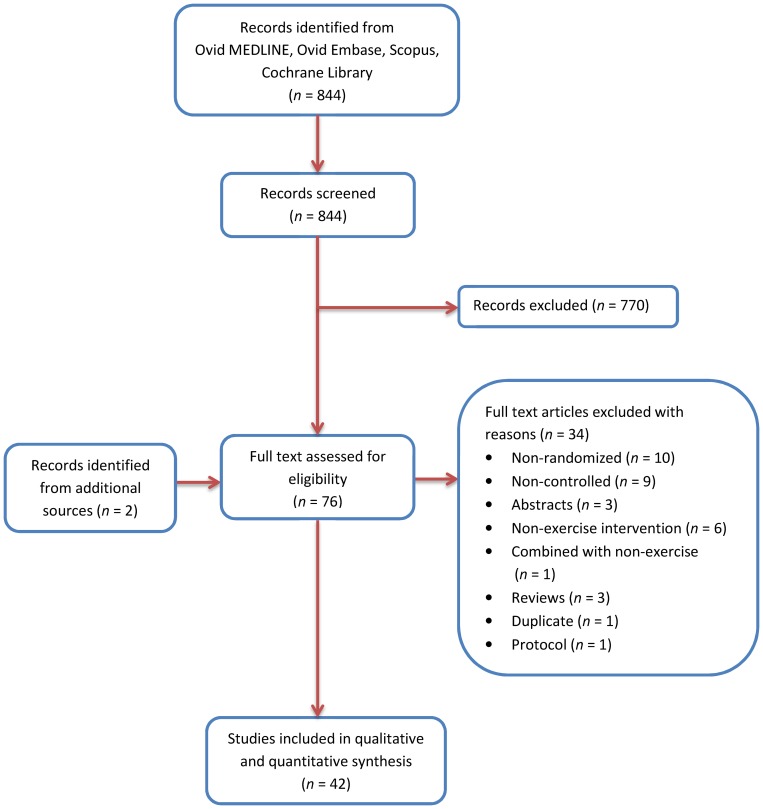
Flow diagram of the process used in selection of the randomized controlled trials included in this systematic review and meta-analysis.

The process followed in the selection of eligible studies is summarized in Figure 1. After full text examination, 42 randomized controlled trials (RCT) were included in the final analysis.

### Study characteristics

Characteristics of the studies included in the systematic review and meta-analysis are summarized in Tables 1 and 2. Further characteristics of the included studies are also provided in Tables S1 and S2. The total number of participants was 1627 (801 males, 826 females) with a median sample size of 24 participants per study (range 10–114). Participant age ranged from 19 to 72 years (median 47 years). Eleven studies included females only [13], [24]–[33] while 9 studies included males only [34]–[43]. The median duration of the studies was 12 weeks (range: 4–52 weeks). The quality of the included studies ranged from 2–5 with a median quality score of 3. The study design comprised 40 parallel [12]–[14], [24], [25], [27]–[61] and 2 crossover studies [26], [62]. Some of the articles included results from separate, independent trials testing the effects of two or more exercise modalities on arterial stiffness so that the 42 articles yielded a total of 46 trials that measured PWV (20 aerobic, 14 resistance and 12 combined) and 23 trials that measured AIx (16 aerobic, 5 resistance and 2 combined) for the final analysis. Of the 23 trials that measured AIx, only 6 studies reported heart rate-adjusted AIx [12], [31], [32], [34], [46], [53]. The methods for quantification of PWV differed between studies and included 28 trials carotid-femoral (cf-PWV), 17 trials brachial-ankle (ba-PWV) and 1 trial femoral-ankle (fa-PWV) measurements. The relative intensity of exercise ranged from 55% to 100% with a median of 74% HRmax while the median of absolute intensity was 7.2 (range 4–12 METs). The duration of exercise sessions ranged from 20 to 100 minutes (median: 40 minutes) and the frequency from 1 to 6 sessions per week (median: 3 per week).

**Table 1:** Characteristics of aerobic exercise studies included in the systematic review and meta-analysis.

Author	Health Status	Sample size	Age (years)	Male%	Basal BMI (kg/m^2^)	Basal SBP (mmHg)	Basal DBP (mmHg)	Type of Exercise	Intensity	Session duration (minutes)	Frequency of sessions	Duration of Intervention (weeks)	Jadad's Score
Beck et al. 2013 [12]	Prehypertension	28	20.1±0.9	68	28.7±1.4	132±3	81±1	W/R	65–85%	45	3	8	3
Choi et al. 2012 [24]	Diabetes II	75	53.8±7.2	0		120 (118–130)	80 (79–80)	W	3.6–6 MET	60	5	12	5
Ciolac et al. 2010 AIT [25]	Risk of Hypertension	17	24.4±3.8	0	23.5±4.8	106±9.9	65±9.5	W/R	80–90%peakO2	40	3	16	3
Ciolac et al. 2010 CMT [25]	Risk of Hypertension	17	26.6±4.9	0	24.3±4.6	106±8.3	64.9±6.8	W/R	60–70%peakO2	40	3	16	3
Ferrier et al. 2001[26]	Systolic hypertension	10	64±7	50	29.5±1.3	154±7		C	65%Hrmax	40	3	8	3
Goldberg et al. 2012 [35]	Risk of Hypertension	30	20.5±0.5	100	23.3±0.5	115±1.4	69±1.6	C	65%Hrmax	30	3	4	2
Gumaraes et al. 2010 AIT [44]	Hypertension	22	45±9	77	29±5	125±9	81±5	R	65%Hrmax	40	3	16	5
Gumaraes et al. 2010 CMT [44]	Hypertension	21	50±8	62	28±4	124±9	81±9	R	65–80%Hrmax	40	3	16	5
Heydari et al. 2013 [36]	Healthy	34	24.4±4.7	100	28.4±0.6	120±2.4	64±1.8	C	80–90%Hrmax	20	3	12	2
Heydari et al. 2013 [37]	Healthy	38	26.5	100	28.4±2.4	119.6±9.9	63.7±7.3	C	80–90%Hrmax	20	3	12	5
Ho et al. 2012 [14]	Overweight/Obese	31	55 (44–62)	13	33 (25–46)	120 (96–159)	67 (55–86)	W	60%Hrr	30	5	12	4
Kearney et al. 2014 [45]	Overweight	65	45±6.2	77	29.2±4	122±12	85±7.4	W			5	26	4
Koh et al. 2010 ID [46]	CKD	23	52.3±10.9	61	27.6±7	148±22	82±10	C	12–13 Borg	30–45	3	26	2
Koh et al. 2010 HB [46]	CKD	23	52±14	65	27.9±4.9	143±32	78±16	W	12–13 Borg	30–45	3	26	2
Krustrup et al. 2010 FG [27]	Premenopausal	28	37±2	0	25±0.9	113±2	74±2	FB	80–84%Hrmax	60	2	16	3
Krustrup et al. 2010 RG [27]	Premenopausal	24	37±1	0	23.7±0.7	112±2	71±2	R	80–84%Hrmax	60	2	16	2
Krustrup et al. 2013 [38]	Hypertension	33	46	100	30±3.3	151	92	Soccer	85–90%Hrmax	60	2	26	2
Madden et al. 2009 [47]	Diabetes II	34	71.4	53	30±1.1	150±6	83±2	C	65–80%Hrmax	40	3	13	5
Madden et al. 2013 [48]	Diabetes II	52	68.5±0.9	58	30.9±1	148±4	82±3	C	60–75%Hrmax	40	3	26	4
Mustata et al. 2011 [49]	CKD	18	64 (55–73)	67	28 (25–32)	140 (130–150)	78 (70–80)	W/C	12–50 Borg	20–60	4	52	4
Nualnim et al. 2011 [50]	Hypertension	43	58±2	26	29±1	131±3	76±2	Swimming	60–75%Hrmax	20–45	3.5	12	4
Oudegeest-Sander et al. 2013 [51]	Healthy	22	68±3	50	27±2.6	131±9	76±6	C	70–85%Hrmax	30	3	52	4
Parnell et al. 2002 [52]	CHF	21	57±15	90	26±0.7	106±7	62±2	W/C	50–60%Hrmax	30–60	6	8	2
Sugawara et al. 2012 [31]	Post menopause	22	59±2	0	23.4±1	117±3	71±2	C	60–75%Hrmax	25–45	4.5	8	5
Toussaint et al. 2008 [62]	CKD	18	67 (60–83)	50	27±4	144±16	75±12	C		30	3	12	4
Westhoff et al. 2008 [53]	Hypertension	24	66.1±4	46	28.6±4.4	134±20	73±21.6	C	2±0.5 lactate	20	3	12	4
Yoshizawa et al. 2009 [33]	Healthy	24	47±2	0	24.6±1.1	120±3	74±3	C	60–70%peakO2	30	2	12	2

AIT, aerobic interval training; C, cycling; CHF, congestive heart failure; CKD, chronic kidney disease; CMT, continuous moderate-intensity training; HB, home-based; HRmax, maximum heart rate; Hrr, heart rate reserve; FB, football; FG, football group; ID, intradialytic; MET, metabolic equivalents; R, running; RG, running group; W, walking.

**Table 2:** Characteristics of resistance/combined exercises studies included in the systematic review and meta-analysis.

Author	Health Status	Sample size	Age (Years)	Male%	Body weight (kg)	Basal BMI (kg/m^2^)	Basal SBP (mmHg)	Basal DBP (mmHg)	Intensity	Session duration (minutes)	Frequency of Sessions	Duration of Intervention (weeks)	Jadad's Score
**Resistance exercise**													
Beck et al. 2013 [12]	Prehypertension	30	21.1±0.6	70	84.2±4.7	27.4±1.3	130.4±3	80±3	60% 1RM	45	3	8	3
Cortez-Cooper et al. 2008 [54]	Healthy	25	52±2	28	76±3	26.8±1.1	113±3	66±2	70% 1RM	30–45	3	13	2
Croymans et al. 2014 [34]	Overweight/Obese	36	21.5	100	97	31	132	81	100%1RM	60	3	12	3
Heffernan et al. 2013 [56]	Prehypertension	21	60±2	29		24±1	140±4	83±2	40–60%1RM		3	12	3
Ho et al. 2012 [14]	Overweight/Obese	32	52 (43–59)	12.5	89	33	126	71	70% 1RM	30	5	12	4
Miyachi et al. 2004 [40]	Healthy	28	22±1	100	66.5±2.4	22.2±0.7	116±3	69±1	80% 1RM	45	3	16	4
Okamoto et al. 2006 ERT [30]	Healthy	15	18.9±0.3	0	55.5±6.1	21.7±2.1	105	61	100%1RM	15	3	8	4
Okamoto et al. 2006 CRT [30]	Healthy	14	19±0.3	0	52.7±7.5	21.9±3.1	102	60	80% 1RM	15	3	8	4
Okamoto et al. 2008 [41]	Healthy	19	19.4	100					40% 1RM		2	8	3
Okamoto et al. 2009 UL [43]	Healthy	15	20±0.4	100	63±13	23±3.6	119±11	67±8	80% 1RM	75	2	10	3
Okamoto et al. 2009 LL [43]	Healthy	15	20±0.5	100	63±12	22±3.6	120±12	68±7	80% 1RM	75	2	10	3
Okamoto et al. 2009 ERT [42]	Healthy	15	19.6	100	62	21	118	66	80% 1RM	105	2	10	3
Okamoto et al. 2009 CRT [42]	Healthy	15	19	100	64	23	117	62	80% 1RM	105	2	10	3
Okamoto et al. 2011 [59]	Healthy	26	18.5	73	65.6±11.5	23.3±4.1	115±13	64±7	50% 1RM	50	2	10	3
Okamoto et al. 2013 ALRT [60]	Healthy	15	19	53	61	23	114	65	50–80%1RM	60	2	10	2
Okamoto et al. 2013 BLRT [60]	Healthy	15	19	47	58	22	115	65	50–80%1RM	60	2	10	2
Yoshizawa et al. 2009 [33]	Healthy	23	47±2	0	59±2	24.8±1	122±7	78±4	60% 1RM	30	2	12	2
**Combined (aerobic and resistance) exercise**													
Cortez-Cooper et al. 2008 [54]	Healthy	24	51±1	29	76±5	27±1.1	118±3	68±2	60–75%Hrr/70% 1RM	30–45	4	13	3
Dobrosielski et al. 2012 [55]	Diabetes II	114	57±6	68	97±2.1	33±0.6	127±1.6	72±1.1	60–90%Hrmax/50% 1RM	60	3	26	3
Figueroa et al. 2011[13]	Postmenopause	24	54±2	0	62	24			60%Hrmax/60% 1RM	40	3	12	3
Ho et al. 2012 [14]	Overweight/Obese	33	53 (43–64)	12	90	33	118	66	60%Hrr/75% 1RM	30	5	12	4
Kawasaki et al. 2011 [57]	Healthy	57	62±0.8	39	60±1.8	23.7±0.6	137±3.2	81±1.6	50%peakO2	100	2	26	3
Loimaala et al. 2009 [39]	Diabetes II	48	53.6±6.2	100	90±9.5	29±3.7	145±2.8		65–75%peakO2/60–80% 1RM	30	4	104	3
Miura et al. 2008 1DW [28]	Postmenopause	41	69±6.5	0	52±6.7	23±2.4	126±14	74±8	70%Hrmax	45	1	12	2
Miura et al. 2008 2DW [28]	Postmenopause	36	69.5±7	0	54±7	24±2.7	123±14	73±9	70%Hrmax	45	2	12	2
Ohta et al. 2012 [29]	Postmenopause	26	72±4	0		23±2.6	151±19	85±9.8	4 METs	20	5	12	3
Okamoto et al. 2007 BRT [58]	Healthy	17	18.5±0.2	65	58.4±3.2	22.1±0.7	113.6±3.4	62.5±2.2	60%Hrmax/80% 1RM		2	8	4
OKamoto et al. 2007 ART [58]	Healthy	16	18.5±0.2	69	59±3	23±2	113.5±4	65±2	60%Hrmax/80% 1RM		2	8	4
Stewart et al. 2005 [61]	Prehypertension	104	63	49	83	29	140	77	60–90%Hrmax/50% 1RM	60	3	26	3
Wong et al. 2014 [32]	Postmenopause	30	57	0	88	34	133	77		50	3	8	4

1DW, 1 day per week, 2DW, 2 days per week; ALRT, low intensity after high intensity resistance training; ART, aerobic training after resistance training; BLRT, low intensity before high intensity resistance training; BRT, aerobic training before resistance training; CRT, concentric resistance training; ERT, eccentric resistance training; Hrmax, maximum heart rate; LL, lower limb; MET, metabolic equivalent; UL, upper limb; RM, repetition maximum;

### Aerobic exercise and PWV

**Figure 2:**
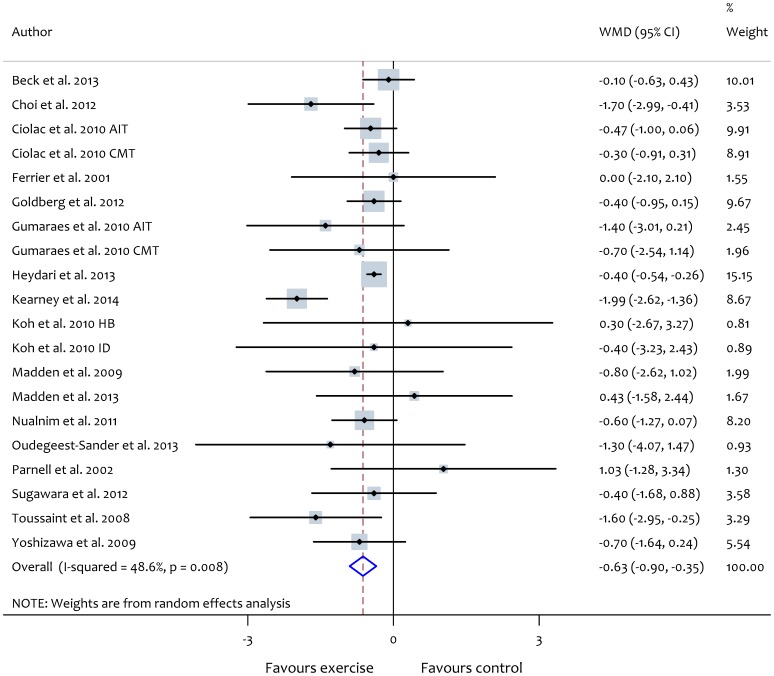
Forest plot showing the effect of aerobic exercise on pulse wave velocity (PWV). AIT, aerobic interval training; CMT, continuous moderate-intensity training; ID, intradialytic; HB, home-based.

**Figure 3:**
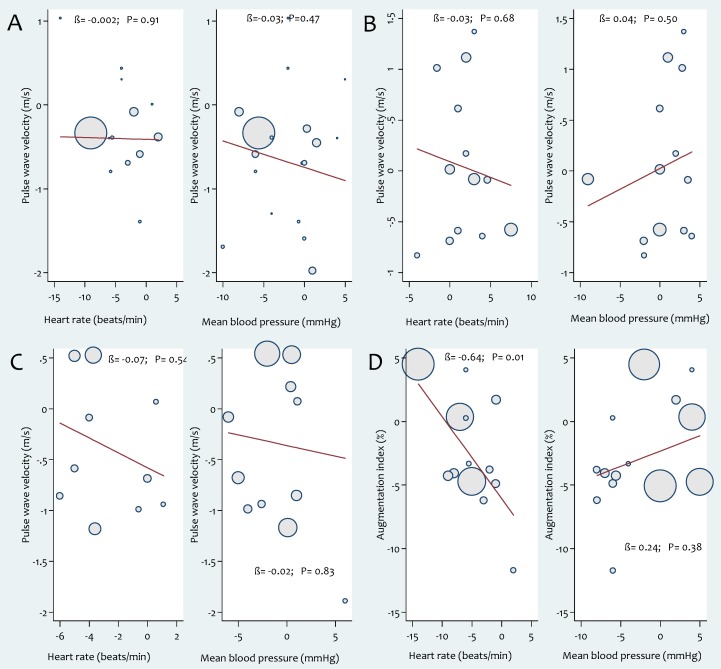
Associations between pulse wave velocity (PWV) and changes in heart rate and mean arterial blood pressure in response to (a) aerobic (b) resistance (c) combined aerobic and resistance exercise intervention. (d) Association between augmentations index (AIx) and changes in heart rate and mean arterial blood pressure in response to aerobic exercise intervention. Each study is depicted by a circle where the circle size represents the degree of weighting for the study based on participant numbers in the study.

Data synthesis from aerobic exercise trials revealed a significant reduction in PWV with exercise (WMD: −0.63 m/s, 95% CI: −0.90, −0.35, *P*<0.01). However, these studies were characterised by significant heterogeneity (*X^2^* = 36.9, *P*<0.01, *I^2^* = 48.6%) (Figure 2). Aerobic exercise training showed significantly greater reduction in ba-PWV in comparison with cf-PWV (Table 3). Subgroup analysis showed significantly greater reduction in PWV after aerobic exercise intervention in participants with stiffer arteries (PWV >8 m/s) (Table 3). In addition, there was a tendency for larger reductions in arterial stiffness with aerobic exercise in studies lasting more than 10 weeks (Table 3). The outcomes of subgroup analyses according to participant health status are reported in Table 4. Meta-regression analyses did not show evidence of a relationship between exercise characteristics and PWV (Figure S1) and changes in PWV were not related to changes in heart rate or mean blood pressure after the interventions (Figure 3).

**Table 3:** Subgroup analyses of the effects exercise modalities on pulse wave velocity (PWV) using a random-effects model.

	Aerobic Exercise			Resistance Exercise			Combined (aerobic and resistance)		
	Trials (No.)^a^	WMD% (95% CI)	*P* value^b^	Trials (No.)^a^	WMD% (95% CI)	*P* value^b^	Trials (No.)^a^	WMD% (95% CI)	*P* value^b^
Outcome (m/s)			0.03			0.89			0.29
Carotid-femoral PWV	17 (422)	−0.39 (−0.52 to −0.27)		6 (144)	−0.12 (−0.67 to 0.43)		5 (320)	−0.07 (−0.66 to 0.51)	
Brachial-ankle PWV	10 (377)	−1.01 (−1.57 to −0.44)		12 (217)	−0.07 (−0.51 to 0.37)		8 (247)	−0.58 (−1.16 to 0.01)	
Age of participants (years)			0.96			0.36			0.99
≤ 50 years	9 (262)	−0.62 (−0.98 to −0.26)		13 (253)	0.001 (−0.39 to 0.39)		2 (33)	−0.35 (−2.01 to 1.31)	
> 50 years	11 (343)	−0.67 (−1.11 to −0.24)		1 (25)	−0.84 (−2.04 to 0.36)		10 (504)	−0.27 (−0.73 to 0.18)	
Gender			0.53			0.71			0.75
Female	5 (155)	−0.52 (−0.87 to −0.18)		3 (52)	0.09 (−0.81 to 1.01)		5 (157)	−0.47 (−1.14 to 0.19)	
Male	2 (68)	−0.40 (−0.53 to −0.26)		6 (115)	−0.14 (−0.74 to 0.45)		1 (48)	−0.70 (−1.77 to 0.37)	
Body mass index (kg/m^2^)			0.47			0.15			0.35
≤ 24.9	5 (110)	−0.42 (−0.72 to −0.13)		10 (168)	0.20 (−0.25 to 0.66)		7 (217)	−0.57 (−1.26 to 0.11)	
≥ 25.0	14 (420)	−0.68 (−1.13 to −0.24)		3 (91)	−0.41 (−0.80 to −0.03)		5 (320)	−0.07 (−0.66 to 0.51)	
Baseline systolic pressure (mmHg)			0.27			0.16			0.67
≤ 120	8 (244)	−0.41 (−0.54 to −0.28)		10 (170)	0.20 (−0.27 to 0.67)		3 (57)	−0.50 (−1.67 to 0.67)	
> 120	12 (361)	−0.81 (−1.38 to −0.23)		3 (89)	−0.40 (−0.75 to −0.06)		8 (456)	−0.16 (−0.68 to 0.35)	
Baseline diastolic pressure (mmHg)			0.39			0.29			0.24
≤ 80	12 (350)	−0.43 (−0.55 to −0.30)		12 (223)	0.10 (−0.29 to 0.50)		8 (382)	−0.17 (−0.76 to 0.41)	
> 80	7 (245)	−0.80 (−1.71 to 0.10)		1 (36)	−0.60 (−1.09 to −0.10)		2 (83)	−1.21 (−2.54 to 0.11)	
Baseline PWV (m/s)			0.01						
≤ 8	6 (151)	−0.37 (−0.50 to −0.25)		4 (96)	0.07 (−0.60 to 0.76)		-	-	
> 8	14 (454)	−1.00 (−1.43 to −0.57)		10 (182)	−0.10 (−0.59 to 0.38)		12 (537)	−0.35 (−0.82 to 0.12)	
Intensity of exercise (METs)			0.51			0.63			0.59
≤ 7	10 (321)	−0.52 (−0.87 to −0.17)		6 (128)	−0.15 (−0.75 to 0.44)		7 (402)	−0.19 (−0.79 to 0.42)	
> 7	8 (201)	−0.39 (−0.52 to −0.26)		8 (150)	0.05 (−0.49 to 0.59)		4 (105)	−0.54 (−1.42 to 0.34)	
Duration of the study (weeks)			0.09			0.11			0.87
≤ 10	5 (111)	−0.21 (−0.57 to 0.13)		11 (194)	0.12 (−0.31 to 0.56)		3 (63)	−0.28 (−1.43 to 0.87)	
> 10	15 (494)	−0.80 (−1.16 to −0.44)		3 (84)	−0.62 (−1.03 to −0.22)		9 (474)	−0.33 (−0.85 to 0.18)	
Quality of studies (Jadad score)			0.37			0.86			0.91
< 3	5 (121)	−0.39 (−0.85 to 0.05)		4 (78)	−0.008 (−0.87 to 0.85)		2 (77)	−0.43 (−1.65 to 0.79)	
≥ 3	15 (484)	−0.70 (−1.04 to −0.36)		10 (200)	−0.08 (−0.50 to 0.34)		10 (460)	−0.35(−0.87 to 0.17)	

a Number of trials or subgroups (number of participants)

b *P* value for the meta-regression analyses between subgroups,

PWV pulse wave velocity, WMD weighted mean difference.

**Table 4:** Subgroup analyses of the effect exercise modalities (aerobic, resistance or combined) on indices of arterial stiffness (pulse wave velocity and augmentation index) according to the health status of the included participants.

	Trials (No.)^a^	WMD% (95% CI)	*P* value
**Aerobic exercise (Pulse wave velocity)**			
Healthy	3 (84)	−0.41 (−0.55 to −0.27)	< 0.01
Overweight/obese	1 (65)	−1.99 (−2.62 to −1.36)	< 0.01
Post-menopause	1 (22)	−0.40 (−1.68 to 0.88)	0.54
Pre-hypertension	4 (28)	−0.32 (−0.59 to −0.04)	0.03
Hypertension	4 (86)	−0.66 (−1.23 to −0.10)	0.02
Diabetes II	3 (161)	−0.89 (−2.09 to 0.32)	0.15
Congestive heart failure	1 (21)	1.03 (−1.28 to 3.34)	0.38
Chronic kidney disease	3 (64)	−1.14 (−2.26 to −0.01)	0.05
**Aerobic exercise (Augmentation index)**			
Healthy	2 (72)	−4.30 (−8.58 to −0.02)	0.05
Overweight/obese	1 (31)	1.57 (−4.75 to 7.89)	0.63
Pre-menopause	2 (52)	−2.55 (−7.55 to 2.44)	0.32
Post-menopause	1 (22)	−3.40 (−15.12 to 8.32)	0.57
Pre-hypertension	2 (28)	−7.16 (−14.79 to 0.46)	0.07
Hypertension	3 (100)	−4.60 (−9.44 to 0.24)	0.06
Congestive heart failure	1 (21)	4.07 (2.27 to 5.87)	< 0.01
Chronic kidney disease	4 (82)	−4.28 (−7.34 to −1.22)	< 0.01
**Resistance exercise**			
Healthy	12 (212)	0.03 (−0.43 to 0.47)	0.91
Overweight/obese	1 (36)	−0.60 (−1.09 to −0.11)	0.72
Pre-hypertension	1 (30)	−0.10 (−0.65 to 0.45)	0.02
**Combined (resistance and aerobic) exercise**			
Healthy	4 (114)	−0.59 (−1.55 to 0.37)	0.23
Post-menopause	5 (157)	−0.48 (−1.15 to 0.19)	0.16
Pre-hypertension	1 (104)	0.50 (−0.02 to 1.02)	0.06
Diabetes II	2 (162)	−0.32 (−1.19 to 0.55)	0.47

a Number of trials or subgroups (number of participants).

Visual inspection of the funnel plot for the aerobic exercise studies (Figure S2) together with Egger's regression test (β: −0.40, *P* = 0.31) suggest low likelihood of publication bias.

### Resistance exercise and PWV

**Figure 4:**
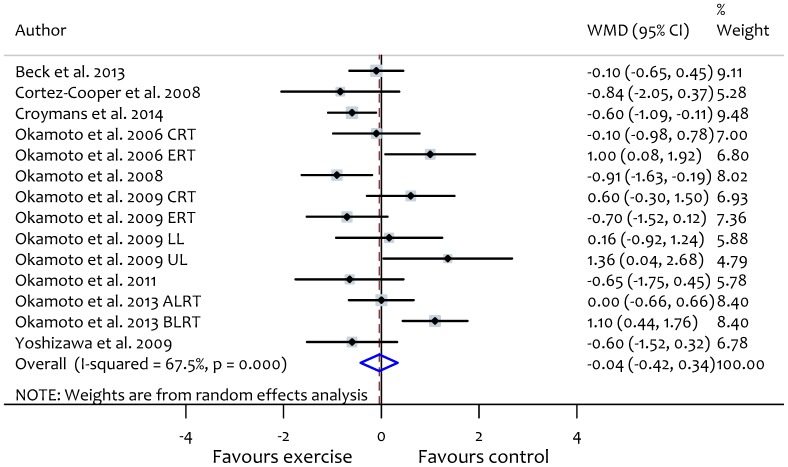
Forest plot showing the effect of resistance exercise on pulse wave velocity (PWV). ERT, eccentric resistance training; CRT, concentric resistance training; UL, upper limb; LL, lower limb; ALRT, low intensity after high intensity resistance training; BLRT, low intensity before high intensity resistance training.

Overall, resistance exercise training was ineffective in improving PWV (WMD: −0.04 m/s, 95% CI: −0.42, 0.34, *P* = 0.82) but there was significant heterogeneity between studies (*X^2^* = 39.9, P<0.01, *I^2^* = 67.5) (Figure 4). Subgroup analyses of resistance exercise studies showed no significant differences between groups based on participant or study characteristics (Table 3) and meta-regression analyses found no evidence for relationships between resistance exercise characteristics and arterial stiffness (Figure S3). Subgroup analyses according to participant health status are reported in Table 4. Changes in PWV were not related to changes in heart rate or blood pressure after the interventions (Figure 3).

Visual inspection of the funnel plot did not show evidence of publication bias for the resistance exercise studies included in the meta-analysis (Figure S2) and Egger's regression test outcomes (β: −0.54, *P* = 0.50) confirmed the likely absence of publication bias.

### Combined (aerobic and resistance) exercise and PWV

**Figure 5:**
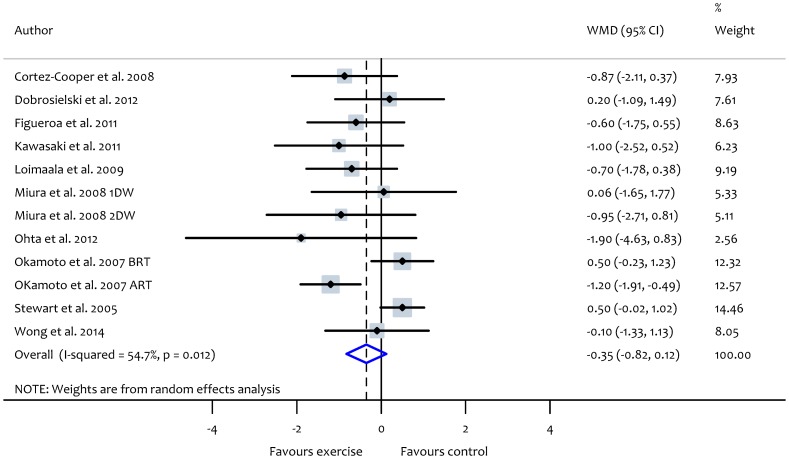
Forest plot showing the effect of combined (aerobic and resistance) exercise on pulse wave velocity (PWV). 1DW, 1 day per week, 2DW, 2 days per week; BRT, aerobic training before resistance training, ART, aerobic training after resistance training.

Analysis of data from combined modalities exercise trials showed a non-significant reduction in PWV (WMD: −0.35 m/s, 95% CI: −0.82, 0.12, *P* = 0.15) with significant heterogeneity between studies (*X^2^* = 24.3, *P*<0.01, *I^2^* = 54.7) (Figure 5). Subgroup analyses of combined (aerobic and resistance) exercise studies demonstrated no significant differences between groups based on participant characteristics (Table 3). The outcomes of subgroup analyses according to participant health status are summarized in Table 4. Meta-regression analyses showed no significant relationships between characteristics of the combined exercise interventions and PWV (Figure S4). Changes in PWV were not related to changes in heart rate or mean blood pressure after the interventions (Figure 3).

Neither funnel plot (Figure S2) nor Egger's regression test (β: 0.60, *P* = 0.10) suggested evidence of publication bias for the studies included in the combined exercise meta-analysis.

### Effect of exercise modalities on AIx%

**Figure 6:**
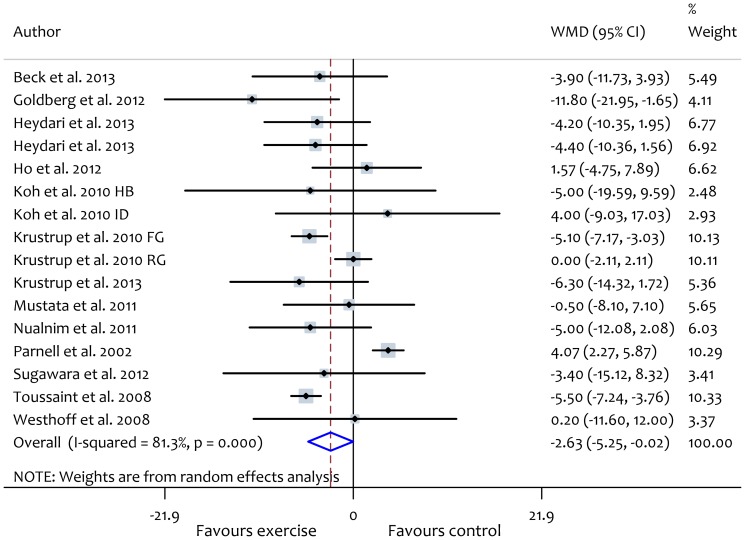
Forest plot showing the effect of aerobic exercise on augmentation index (AIx). intradialytic; HB, home-based; FG, football group, RG, running group.

**Figure 7:**
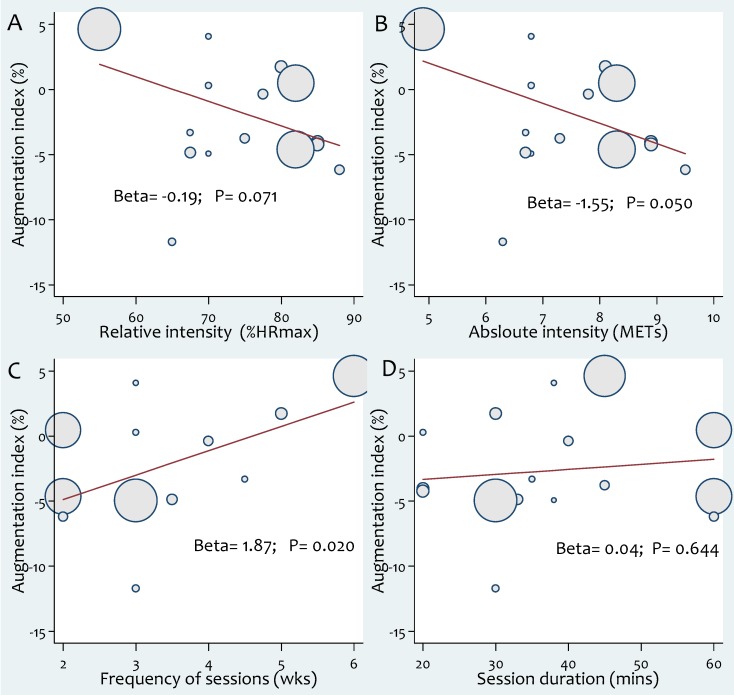
Associations between aerobic exercise intervention characteristics and augmentation index (AIx): (a) relative intensity; (b) absolute intensity; (c) session frequency; (d) session duration. Each study is depicted by a circle where the circle size represents the degree of weighting for the study based on participant numbers in the study.

Aerobic exercise intervention lowered the AIx significantly (WMD: −2.63%, 95% CI: −5.25 to −0.02, *P* = 0.05) (Figure 6). There was significant heterogeneity between studies (*X^2^* = 80.3, *P*<0.01, *I^2^* = 81.3) but subgroup analyses did not reveal evidence of significant differences between groups based on participant or study characteristics (Table S3). Subgroup analyses according to participant health status are reported in Table 4. Meta-regression analyses showed that frequency of aerobic exercise sessions/week was significantly associated with an increase in (worsened) AIx (β: 1.87, CI 0.34, 3.39, *P* = 0.02). Conversely, levels of absolute (β: −1.55, CI −3.09, 0.0001, *P* = 0.05) and relative (β: −0.19, CI: −0.40, 0.02, *P* = 0.07) exercise intensity were inversely associated with AIx (Figure 7). There was no evidence of publication bias in the included studies based on a funnel plot (Figure S2) and egger's regression test (β: −1.14, *P* = 0.61). Changes in AIx correlated significantly with changes in heart rate (β: −0.64, CI: −1.11, −0.17, *P* = 0.011) but not with changes in mean blood pressure (Figure 3).

**Figure 8:**
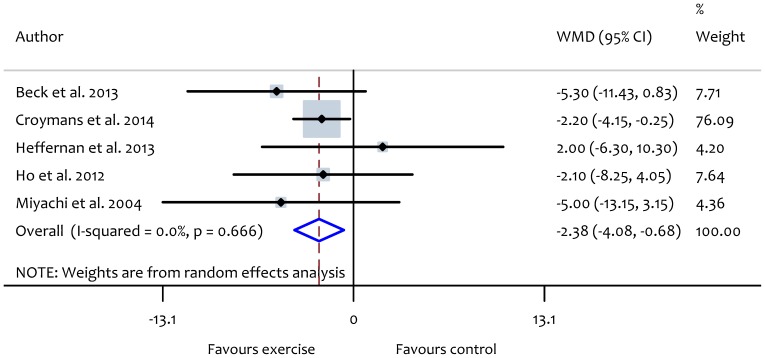
Forest plot showing the effect of resistance exercise intervention on augmentation index (AIx).

Resistance exercise interventions did not reduce AIx significantly (WMD: −1.69%, 95% CI: −4.11 to 0.72, *P* = 0.17) (Figure 8). Furthermore, only two studies [14], [32] investigated the effect of combined aerobic and resistance exercise on AIx and these showed non-significant improvement (WMD: −1.66%, 95% CI: −6.18 to 2.86, *P* = 0.47). Because of the small number of studies which addressed the effect of resistance (5 trials) or combined (2 trials) exercise on AIx, we were unable to perform subgroup or meta-regression analyses to investigate the potential modifying effects of participant or exercise characteristics on changes in AIx.

## Discussion

In the current meta-analysis we observed a significant improvement in PWV and AIx in response to aerobic exercise intervention. This aerobic exercise effect tended to be greater in peripheral (ba-PWV) rather than in central (cf-PWV) indices. Additionally, larger effects observed in participants with more arterial stiffness at baseline and in trials with longer duration. Furthermore, intensity (relative and absolute) rather than volume of exercise (frequency and duration of sessions) was positively associated with improvement in AIx. However, resistance and combined (aerobic and resistance) exercise interventions produced no beneficial effects on PWV or AIx.

Oxidative stress and inflammation are the main insults causing stiffening of the vascular wall [63], [64]. Fragmentation of elastin, deposition of collagen, and smooth muscle proliferation are the end result of continuous exposure to free radicals and inflammatory cytokines [65]. In addition, reduced nitric oxide (NO) and increased vasoconstrictor agents (angiotensin, endothelin, prostaglandins) exacerbate arterial stiffness [66]. Evidence from both animal and human studies shows the beneficial effect of physical activity (exercise) on vascular compliance and remodelling [67]. For example, the arteries of exercised animals had higher elastin and lower collagen than those from physically inactive animals [68]. Moreover, there is evidence that chronic exercise has anti-oxidative effects through up-regulation of superoxide dismutases (SOD1, SOD3) and down-regulation of NAD(P)H oxidase [69]. Furthermore, clinical investigations have documented the anti-inflammatory effect of regular physical activity by increasing the anti-inflammatory cytokines (interleukin 4, 10) and reducing pro-inflammatory cytokines (interleukin 6 and tumour necrosis factor alpha) [70]. Lastly, there is evidence that exercise enhances production of NO and reduces the concentrations of vasoconstrictor agents (endothelin I, angiotensin II) [71].

In our meta-analysis, aerobic exercise reduced PWV and AIx significantly. This finding is in contrast with findings from recently published meta-analyses which did not find a significant positive effect of aerobic exercise in pre-hypertensive, hypertensive and obese participants [72], [73]. These 2 meta-analyses pooled the results from various measures of arterial stiffness and of wave reflection as standardised mean differences. Furthermore, they included non-randomized controlled trials in their analysis. In the present meta-analysis we included only studies that reported the commonly used measures of arterial stiffness and wave reflection (PWV and AIx) [74], [75] and we restricted our analysis to RCTs. Furthermore, we analysed the data as weighted mean difference so that the effect sizes obtained will be clinically applicable [76]. In contrast to the meta-analysis conducted by Montero et al. [72], we observed significant improvement in PWV with aerobic exercise intervention in pre- and hypertensive participants but we suggest that these results are interpreted cautiously because of the small number of studies included in the subgroup analyses (Table 4).

The magnitude of the effect of aerobic exercise training on PWV was greater in peripheral (ba-PWV) rather than in central (cf-PWV) indices of arterial stiffness. This difference might be explained by the apparently-greater shear stress-enhanced release of NO in the peripheral exercising limbs and the nitric oxide-producing small conduit arteries [4], [77].

In the present study, we observed a tendency for a positive dose-response relationship between exercise intensity and improvement in AIx. Previous studies demonstrated that AIx is inversely associated with blood pressure and with peripheral vascular resistance [78]. Exercising with higher intensity might result in augmented release of NO as a consequence of greater shear stress on the vascular endothelium. This augmented release of the vasodilatory and protective molecule NO may leads to greater reduction in peripheral vascular resistance and, eventually, AIx [79]. These findings support the emerging evidence that high intensity physical activity may be more beneficial for cardiovascular health than low intensity exercise and PA guidelines published by the American College of Sports Medicine emphasise the benefit of high intensity exercise for maintaining and improving cardiovascular health [80], [81]. Furthermore, we observed significant correlations between changes in AIx and changes in heart rate after aerobic exercise intervention. Previous studies have shown that AIx is influenced by changes in heart rate or blood pressure [78]. Unfortunately, in the present meta-analysis, only 6 studies reported the measurement of heart-rate adjusted AIx [12], [31], [32], [34], [46], [53].

Overall, there was no effect, positive or negative, of resistance exercise interventions on PWV and AIx. This is contrary to the findings from a previous meta-analysis which showed that resistance exercise increased arterial stiffness significantly [82]. However, in that meta-analysis, subgroup comparison showed that the increased stiffness was observed only in young participants or with higher intensity resistance exercise. One possible reason for the difference between our findings and those of Miyachi (2013) is our inclusion of more studies which have been published recently [14], [34], [56], [59], [60]. We observed that some forms of resistance exercise were associated with improvement, or at least no deterioration, in arterial stiffness. These included lower intensity rather than high intensity training [59], lower-limb rather than upper-limb training [43], eccentric rather than concentric resistance training [42] and the combination of resistance with aerobic training [58].

Combined aerobic and resistance exercise showed no significant improvement in arterial stiffness. However, the proven beneficial effects of resistance exercise on cardio-metabolic parameters make it an indispensable adjunct to aerobic exercise for those patients with cardio-metabolic disorders [83], [84]. Therefore, future studies should focus on combinations of aerobic exercise with modes of resistance exercise that have beneficial, or at least non-harmful, effects on vascular hemodynamic.

Previous studies showed that every 1 m/s increase in PWV is associated with 12–14% increase in the risk of cardiovascular events and 13–15% increase in the risk of CVD mortality [74], [85]. In the present meta-analysis, we observed that aerobic exercise reduced PWV by 0.63 m/s which may translate into a reduction of 8% in cardiovascular events and 9% in cardiovascular mortality. Furthermore, subgroup analyses suggested that there may be bigger effects on PWV and, consequently, on cardiovascular events and mortality of aerobic exercise in higher risk participants (those with PWV ≥8 m/s at baseline) and with longer duration aerobic exercise interventions (>10 weeks).

Our systematic review and meta-analysis has several limitations. The small sample size of most of the included studies may limit the generalizability of our findings. However, the included studies were characterised as good quality on Jadad's Score (more than 2/3 of the studies had ≥3 out of 5 total score). Furthermore, the participants of the included studies were characterised by diverse health status, age, gender and country of origin which may aid in generalizing our results to the general population. Nevertheless, most of the studies which employed resistance exercise interventions recruited healthy participants (**Table 4**). Therefore, further studies are required to assess the effect of resistance exercise in participants with cardio-metabolic diseases. Finally, our meta-analysis was based on summary data from each of the original studies. We did not have access to individual-level data which would have allowed more extensive adjustment for potential confounding factors.

In summary, aerobic exercise improved indices of arterial stiffness and wave reflection significantly. Aerobic exercise training had a greater effect in peripheral (ba-PWV) rather than in central (cf-PWV) indices of arterial stiffness. The effect of aerobic exercise was enhanced with higher intensity exercise, in participants with higher vascular stiffness and with longer duration studies. Resistance exercise had no overall effect on PWV and AIx. The study showed that some forms of resistance exercise may be associated with beneficial effect on arterial stiffness. Therefore, further studies are required to investigate the effect of these forms of resistance exercise alone, or in combination with aerobic exercise, on indices of arterial stiffness and wave reflection.

## Supporting Information

Figure S1 **Associations between aerobic exercise intervention characteristics and pulse wave velocity: (a) relative intensity; (b) absolute intensity; (c) session frequency; (d) session duration.** Each study is depicted by a circle where the circle size represents the degree of weighting for the study based on participant numbers in the study.
(TIF)

Figure S2 **Funnel plot for publication bias of the effect of (a) aerobic (b) resistance (c) combined aerobic and resistance exercise intervention on pulse wave velocity (PWV).** (d) Funnel plot for publication bias of the effect of aerobic exercise intervention on augmentation index (AIx).
(TIF)

Figure S3 **Associations between resistance exercise intervention characteristics and pulse wave velocity (PWV): (a) relative intensity; (b) absolute intensity; (c) session frequency; (d) session duration.** Each study is depicted by a circle where the circle size represents the degree of weighting for the study based on the number of study participants.
(TIF)

Figure S4 **Associations between combined (aerobic and resistance) exercise intervention characteristics and arterial stiffness measured by pulse wave velocity (PWV): (a) relative intensity; (b) absolute intensity; (c) session frequency; (d) session duration.** Each study is depicted by a circle where the circle size represents the degree of weighting for the study based on the number of study participants.
(TIF)

Table S1 Further characteristics of aerobic exercise intervention studies included in the meta-analysis.
(PDF)

Table S2 Further characteristics of resistance/combined exercise intervention studies included in the meta-analysis.
(PDF)

Table S3 Subgroup analyses of the effects aerobic exercise on augmentation index using a random-effects model.
(PDF)

Checklist S1 PRISMA Checklist 2009.
(DOC)
