# Supplementary figures and images for: Effects of Exercise Modalities on Arterial Stiffness and Wave Reflection: A Systematic Review and Meta-Analysis of Randomized Controlled Trials
Source: PLoS One. 2014 Oct 15;9(10):e110034. doi: 10.1371/journal.pone.0110034 (PMC4198209; doi:10.1371/journal.pone.0110034)

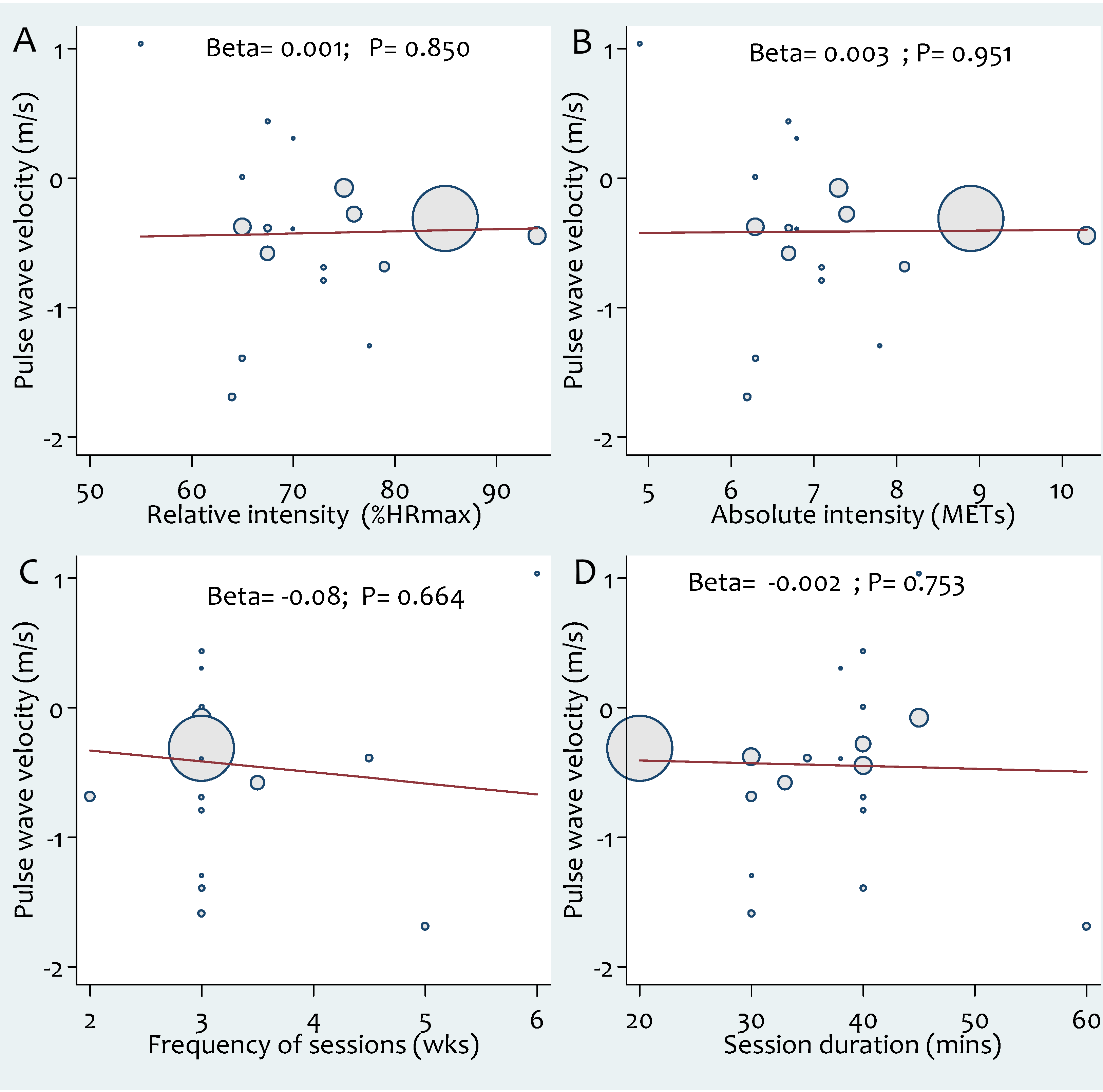

Supplement: Figure S1 — Associations between aerobic exercise intervention characteristics and pulse wave velocity: (a) relative intensity; (b) absolute intensity; (c) session frequency; (d) session duration. Each study is depicted by a circle where the circle size represents the degree of weighting for the study based on participant numbers in the study. (TIF) [file pone.0110034.s001.tif]

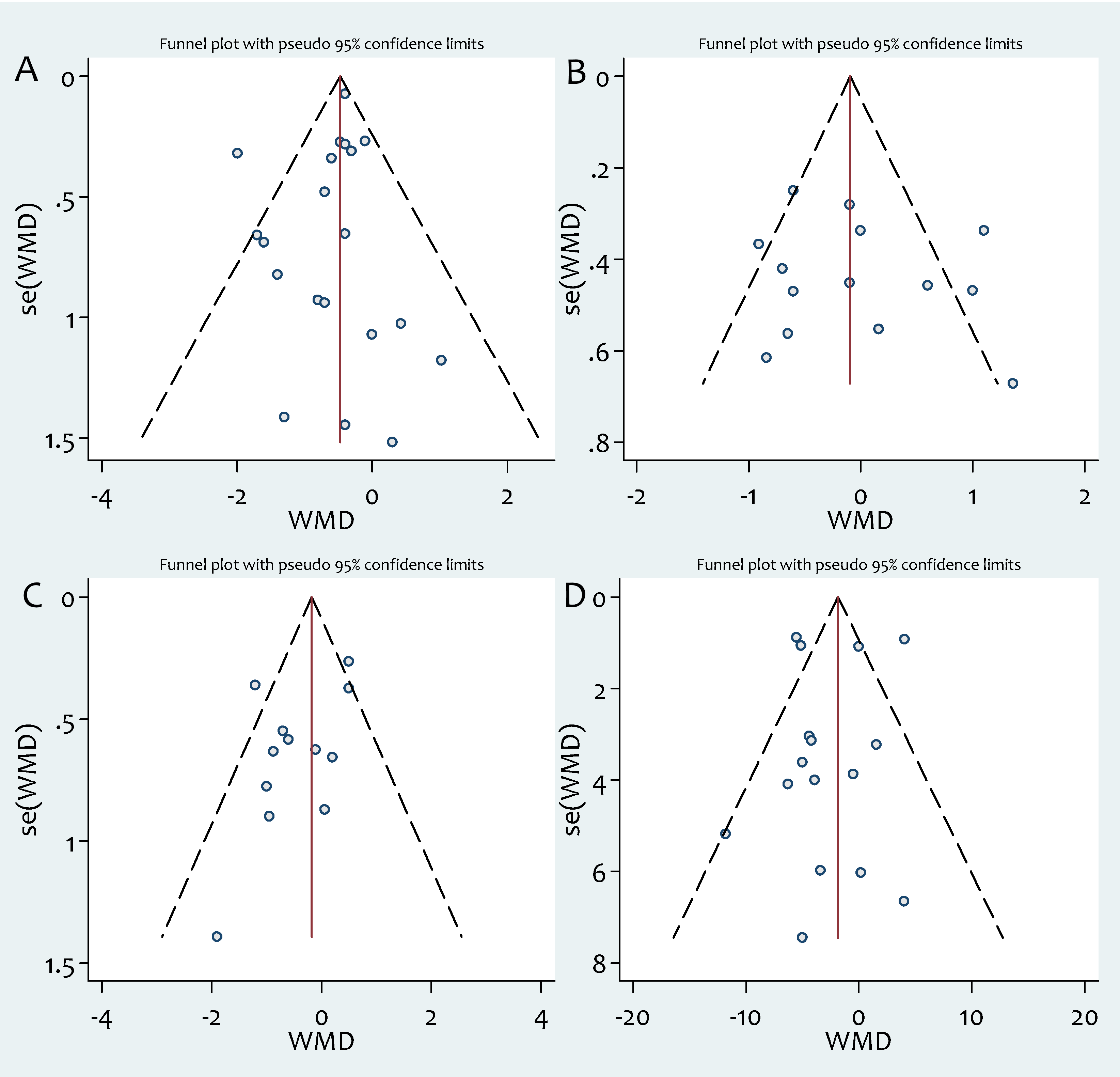

Supplement: Figure S2 — Funnel plot for publication bias of the effect of (a) aerobic (b) resistance (c) combined aerobic and resistance exercise intervention on pulse wave velocity (PWV). (d) Funnel plot for publication bias of the effect of aerobic exercise intervention on augmentation index (AIx). (TIF) [file pone.0110034.s002.tif]

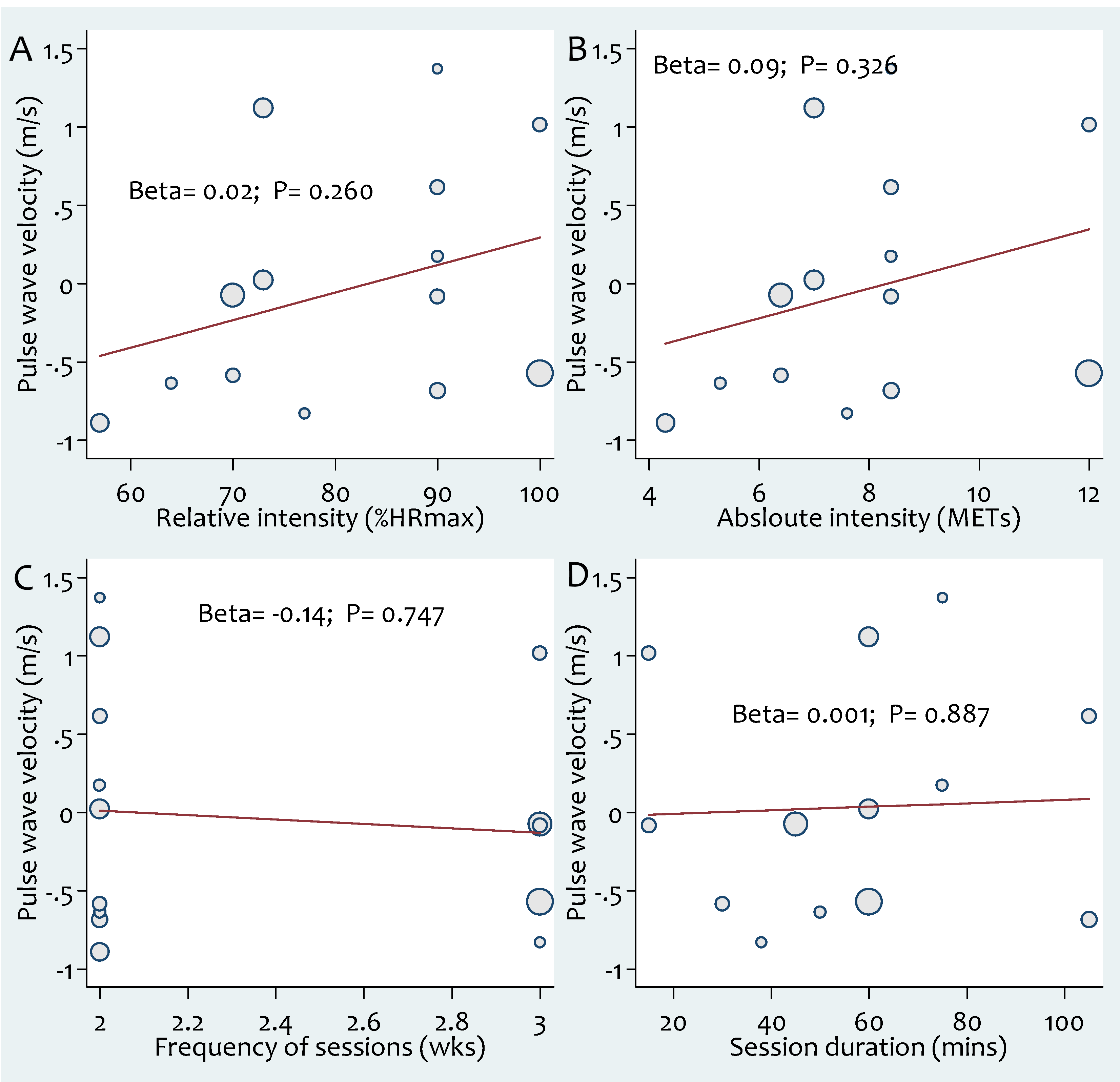

Supplement: Figure S3 — Associations between resistance exercise intervention characteristics and pulse wave velocity (PWV): (a) relative intensity; (b) absolute intensity; (c) session frequency; (d) session duration. Each study is depicted by a circle where the circle size represents the degree of weighting for the study based on the number of study participants. (TIF) [file pone.0110034.s003.tif]

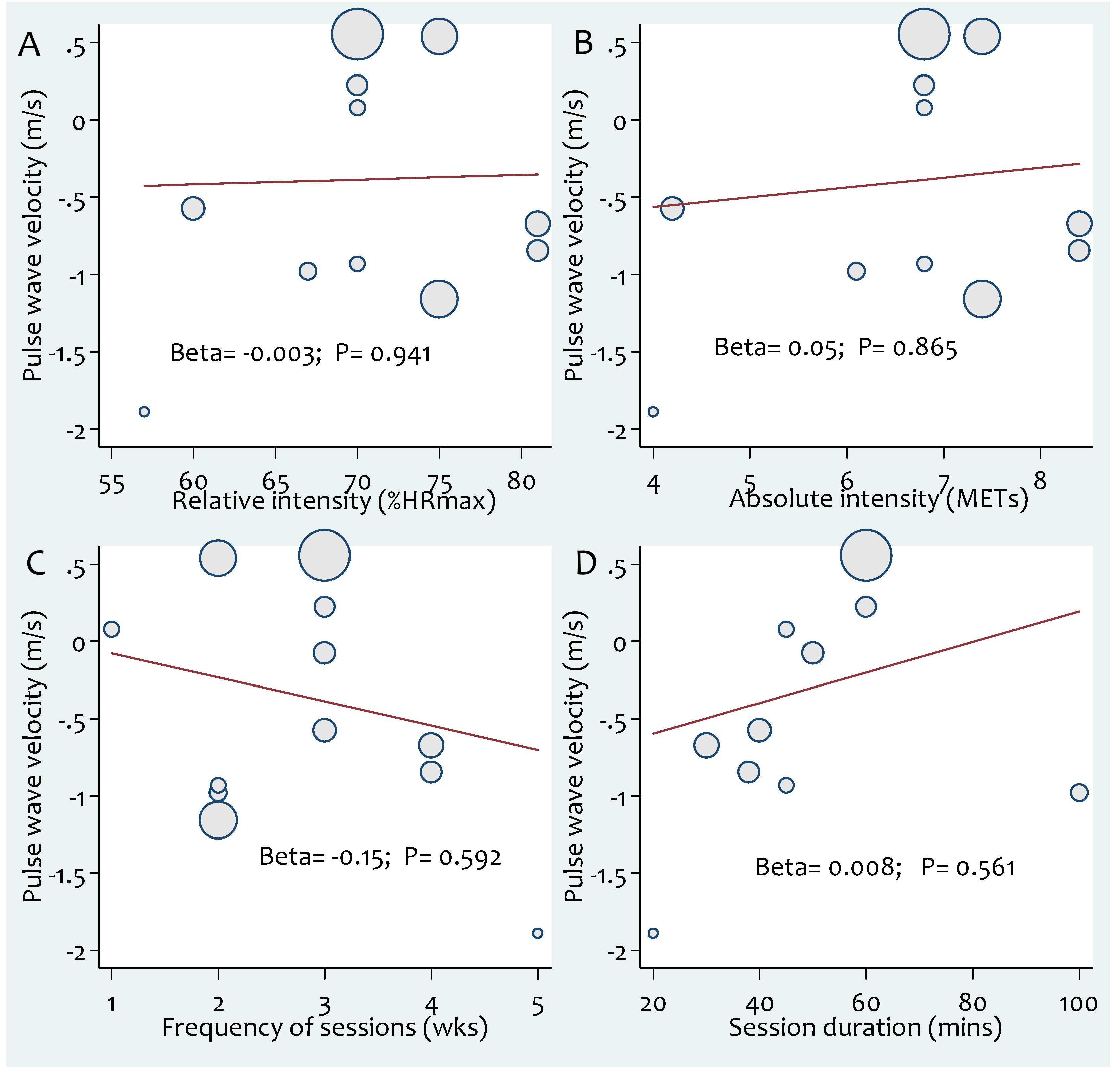

Supplement: Figure S4 — Associations between combined (aerobic and resistance) exercise intervention characteristics and arterial stiffness measured by pulse wave velocity (PWV): (a) relative intensity; (b) absolute intensity; (c) session frequency; (d) session duration. Each study is depicted by a circle where the circle size represents the degree of weighting for the study based on the number of study participants. (TIF) [file pone.0110034.s004.tif]
