# Supplementary material for: Effects of Exercise Modalities on Arterial Stiffness and Wave Reflection: A Systematic Review and Meta-Analysis of Randomized Controlled Trials
Source: PLoS One. 2014 Oct 15;9(10):e110034. doi: 10.1371/journal.pone.0110034 (PMC4198209; doi:10.1371/journal.pone.0110034)
Supplement: Table S1 — Further characteristics of aerobic exercise intervention studies included in the meta-analysis. (PDF) [file pone.0110034.s005.pdf]

Table S1: Further characteristics of aerobic exercise intervention studies included in the meta-analysis

Author	Country	Study Design	Arterial Stiffness	Compliance%	Instrument
Beck et al. 2013 [1]	USA	Parallel	PWV, PWA		SphygmaCor/AT
Choi et al. 2012 [2]	South Korea	Parallel	PWV		Colin Waveform Analyzer
Ciolac et al. 2010 AIT [3]	Brazil	Parallel	PWV	83%	Complior
Ciolac et al. 2010 CMT [3]	Brazil	Parallel	PWV	84%	Complior
Ferrier et al. 2001[4]	Australia	Crossover	PWV, PWA		AT/SPT-301
Goldberg et al. 2012 [5]	Australia	Parallel	PWV, PWA	100%	SphygmaCor/AT
Gumaraes et al. 2010 AIT [6]	Brazil	Parallel	PWV	81%	Complior
Gumaraes et al. 2010 CMT [6]	Brazil	Parallel	PWV	82%	Complior
Heydari et al. 2013 [7]	Australia	Parallel	PWA		SphygmaCor/AT
Heydari et al. 2013 [8]	Australia	Parallel	PWV, PWA		SphygmaCor/AT
Ho et al. 2012 [9]	Australia	Parallel	PWA		SphygmaCor/AT
Kearney et al. 2014 [10]	UK	Parallel	PWV		SphygmaCor/AT
Koh et al. 2010 ID [11]	Australia	Parallel	PWV, PWA	75%	SphygmaCor/AT
Koh et al. 2010 HB [11]	Australia	Parallel	PWV, PWA	71%	SphygmaCor/AT
Krustrup et al. 2010 FG [12]	Denmark	Parallel	PWA	83%	Photoplethysmography
Krustrup et al. 2010 RG [12]	Denmark	Parallel	PWA	82%	Photoplethysmography
Krustrup et al. 2013 [13]	Denmark	Parallel	PWA		Photoplethysmography
Madden et al. 2009 [14]	Canada	Parallel	PWV	90%	Complior
Madden et al. 2013 [15]	Canada	Parallel	PWV	90%	Complior
Mustata et al. 2011 [16]	Canada	Parallel	PWA	80%	SphygmaCor/AT
Nualnim et al. 2011 [17]	USA	Parallel	PWV, PWA	99%	VP-2000/AT/US
Oudegeest-Sander et al. 2013 [18]	The Netherland	Parallel	PWV		Doppler/US
Parnell et al. 2002 [19]	Australia	Parallel	PWV, PWA		Doppler/AT
Sugawara et al. 2012 [20]	Japan	Parallel	PWV, PWA	99%	AT
Toussaint et al. 2008 [21]	Australia	Crossover	PWV, PWA		SphygmaCor/AT
Westhoff et al. 2008 [22]	Germany	Parallel	PWA		CR-2000
Yoshizawa et al. 2009 [23]	Japan	Parallel	PWV		AT

References

1. Beck DT, Martin JS, Casey DP, Braith RW: Exercise training reduces peripheral arterial stiffness and myocardial oxygen demand in young prehypertensive subjects. American Journal of Hypertension 2013, 26(9):1093-1102.
2. Choi KM, Han KA, Ahn HJ, Hwang SY, Hong HC, Choi HY, Yang SJ, Yoo HJ, Baik SH, Choi DS et al: Effects of exercise on sRAGE levels and cardiometabolic risk factors in patients with type 2 diabetes: a randomized controlled trial. Journal of Clinical Endocrinology & Metabolism 2012, 97(10):3751-3758.
3. Ciolac EG, Bocchi EA, Bortolotto LA, Carvalho VO, Greve JM, Guimaraes GV: Effects of high-intensity aerobic interval training vs. moderate exercise on hemodynamic, metabolic and neuro-humoral abnormalities of young normotensive women at high familial risk for hypertension. Hypertension Research - Clinical & Experimental 2010, 33(8):836-843.
4. Ferrier KE, Waddell TK, Gatzka CD, Cameron JD, Dart AM, Kingwell BA: Aerobic exercise training does not modify large-artery compliance in isolated systolic hypertension. Hypertension 2001, 38(2):222-226.
5. Goldberg MJ, Boutcher SH, Boutcher YN: The effect of 4 weeks of aerobic exercise on vascular and baroreflex function of young men with a family history of hypertension. Journal of Human Hypertension 2012, 26(11):644-649.
6. Guimaraes GV, Ciolac EG, Carvalho VO, D'Avila VM, Bortolotto LA, Bocchi EA: Effects of continuous vs. interval exercise training on blood pressure and arterial stiffness in treated hypertension. Hypertension Research - Clinical & Experimental 2010, 33(6):627-632.
7. Heydari M, Boutcher YN, Boutcher SH: The effects of high-intensity intermittent exercise training on cardiovascular response to mental and physical challenge. International Journal of Psychophysiology 2013, 87(2):141-146.
8. Heydari M, Boutcher YN, Boutcher SH: High-intensity intermittent exercise and cardiovascular and autonomic function. Clinical Autonomic Research 2013, 23(1):57-65.
9. Ho SS, Radavelli-Bagatini S, Dhaliwal SS, Hills AP, Pal S: Resistance, aerobic, and combination training on vascular function in overweight and obese adults. Journal of Clinical Hypertension 2012, 14(12):848-854.
10. Kearney TM, Murphy MH, Davison GW, O'Kane MJ, Gallagher AM: Accumulated brisk walking reduces arterial stiffness in overweight adults: evidence from a randomized control trial. Journal of the American Society of Hypertension 2014, 8(2):117-126.
11. Koh KP, Fassett RG, Sharman JE, Coombes JS, Williams AD: Effect of intradialytic versus home-based aerobic exercise training on physical function and vascular parameters in hemodialysis patients: a randomized pilot study. American Journal of Kidney Diseases 2010, 55(1):88-99.
12. Krustrup P, Hansen PR, Randers MB, Nybo L, Martone D, Andersen LJ, Bune LT, Junge A, Bangsbo J: Beneficial effects of recreational football on the cardiovascular risk profile in untrained premenopausal women. Scandinavian Journal of Medicine & Science in Sports 2010, 20 Suppl 1:40-49.
13. Krustrup P, Randers MB, Andersen LJ, Jackman SR, Bangsbo J, Hansen PR: Soccer improves fitness and attenuates cardiovascular risk factors in hypertensive men. Medicine & Science in Sports & Exercise 2013, 45(3):553-560.
14. Madden KM, Lockhart C, Cuff D, Potter TF, Meneilly GS: Short-term aerobic exercise reduces arterial stiffness in older adults with type 2 diabetes, hypertension, and hypercholesterolemia. Diabetes Care 2009, 32(8):1531-1535.
15. Madden KM, Lockhart C, Cuff D, Potter TF, Meneilly GS: Aerobic training-induced improvements in arterial stiffness are not sustained in older adults with multiple cardiovascular risk factors. Journal of Human Hypertension 2013, 27(5):335-339.
16. Mustata S, Groeneveld S, Davidson W, Ford G, Kiland K, Manns B: Effects of exercise training on physical impairment, arterial stiffness and health-related quality of life in patients with

chronic kidney disease: a pilot study. International Urology & Nephrology 2011, 43(4):1133-1141.
17. Nualnim N, Parkhurst K, Dhindsa M, Tarumi T, Vavrek J, Tanaka H: Effects of swimming training on blood pressure and vascular function in adults >50 years of age. In: American journal of cardiology. vol. 109; 2012: 1005-1010.
18. Oudegeest-Sander MH, Olde Rikkert MG, Smits P, Thijssen DH, van Dijk AP, Levine BD, Hopman MT: The effect of an advanced glycation end-product crosslink breaker and exercise training on vascular function in older individuals: a randomized factorial design trial. Experimental Gerontology 2013, 48(12):1509-1517.
19. Parnell MM, Holst DP, Kaye DM: Exercise training increases arterial compliance in patients with congestive heart failure. Clinical Science 2002, 102(1):1-7.
20. Sugawara J, Akazawa N, Miyaki A, Choi Y, Tanabe Y, Imai T, Maeda S: Effect of endurance exercise training and curcumin intake on central arterial hemodynamics in postmenopausal women: pilot study. American Journal of Hypertension 2012, 25(6):651-656.
21. Toussaint ND, Polkinghorne KR, Kerr PG: Impact of intradialytic exercise on arterial compliance and B-type natriuretic peptide levels in hemodialysis patients. Hemodialysis International 2008, 12(2):254-263.
22. Westhoff TH, Schmidt S, Gross V, Joppke M, Zidek W, van der Giet M, Dimeo F: The cardiovascular effects of upper-limb aerobic exercise in hypertensive patients. Journal of Hypertension 2008, 26(7):1336-1342.
23. Yoshizawa M, Maeda S, Miyaki A, Misono M, Saito Y, Tanabe K, Kuno S, Ajisaka R: Effect of 12 weeks of moderate-intensity resistance training on arterial stiffness: a randomised controlled trial in women aged 32-59 years. British Journal of Sports Medicine 2009, 43(8):615-618.
