# Supplementary material for: Effects of Exercise Modalities on Arterial Stiffness and Wave Reflection: A Systematic Review and Meta-Analysis of Randomized Controlled Trials
Source: PLoS One. 2014 Oct 15;9(10):e110034. doi: 10.1371/journal.pone.0110034 (PMC4198209; doi:10.1371/journal.pone.0110034)
Supplement: Table S2 — Further characteristics of resistance/combined exercise intervention studies included in the meta-analysis. (PDF) [file pone.0110034.s006.pdf]

Table S2: Further characteristics of resistance/combined exercise intervention studies included in the meta-analysis

Author	Country	Study Design	Arterial Stiffness	Compliance %	Instrument	Type of Exercise
**Resistance exercise**						
Beck et al. 2013 [1]	USA	Parallel	PWV, PWA	100%	SphygmaCor/AT	Leg extension, leg curl, leg press, lat pull down, chest press, overhead press, biceps curl
Cortez-Cooper et al. 2008 [2]	USA	Parallel	PWV, PWA	87%	US/AT	Seated chest press, horizontal leg press, shoulder press, abdominal crunches, seated hamstring curls, seated row, seated calf raises, low back extension, triceps curls, biceps dumbbell
Croymans et al. 2014 [3]	USA	Parallel	PWV, PWA	99%	SphygmaCor/AT	Dumbbells, squats, cable row, dumbbell front lunge, dumbbell row, dumbbell triceps extension and dumbbell biceps curls, chest press, overhead press, abdominal crunches
Heffernan et al. 2013 [4]	USA	Parallel	PWA	90%	SphygmaCor/AT	Chest press, latissmus dorsi pulldown, back row, biceps curls, leg press, leg extension, leg curl
Ho et al. 2012 [5]	Australia	Parallel	PWA	67-74%	SphygmaCor/AT	Leg press, leg curl, leg extension, bench press, rear deltoid row
Miyachi et al. 2004 [6]	Japan	Parallel	PWA	100%	US/AT	Leg extension, seated chest press, leg curls, lateral rows, squat, and sit-up
Okamoto et al. 2006 ERT [7]	Japan	Parallel	PWV		Form PWV/ABI	Arm curls
Okamoto et al. 2006 CRT [7]	Japan	Parallel	PWV		Form PWV/ABI	Arm curls
Okamoto et al. 2008 [8]	Japan	Parallel	PWV		Form PWV/ABI	Chest press, arm curl, lateral pull down, seated rows, shoulder press, leg extension, leg curls, leg press and sit-up
Okamoto et al. 2009 UL [9]	Japan	Parallel	PWV		Form PWV/ABI	Chest press, arm curls, seated rows, shoulder presses, lat pull down
Okamoto et al. 2009 LL [9]	Japan	Parallel	PWV		Form PWV/ABI	Leg press, squats, seated calf raises, leg extensions and leg curls
Okamoto et al. 2009 ERT [10]	Japan	Parallel	PWV		Form PWV/ABI	Chest press or arm curls, seated rows, leg curls, leg press and sit-up
Okamoto et al. 2009 CRT [10]	Japan	Parallel	PWV		Form PWV/ABI	Chest press or arm curls, seated rows, leg curls, leg press and sit-up
Okamoto et al. 2011 [11]	Japan	Parallel	PWV		Form PWV/ABI	Chest press, arm curls, seated rows, lateral pull down, leg press, leg extension, leg curl and sit-ups
Okamoto et al. 2013 ALRT [12]	Japan	Parallel	PWV		Form PWV/ABI	Chest press, arm curls, seated rows, leg curls, leg press
Okamoto et al. 2013 BLRT [12]	Japan	Parallel	PWV		Form PWV/ABI	Chest press, arm curls, seated rows, leg curls, leg press
Yoshizawa et al. 2009 [13]	Japan	Parallel	PWV		AT	Leg curl, leg press, hip abduction, hip flexion, vertical press and sit-ups
**Combined exercise**						
Cortez-Cooper et al. 2008 [2]	USA	Parallel	PWV, PWA	95%	US/AT	3days: Seated chest press, horizontal leg press, shoulder press, abdominal crunches, seated hamstring curls, seated row, seated calf raises, low back extension, triceps curls, biceps dumbbell and other 2 days walking or cycling
Dobrosielski et al. 2012 [14]	USA	Parallel	PWV	92%	AT	Resistance exercise (multi-station machine) and aerobic exercise cycling or stairstepper
Figueroa et al. 2011[15]	South Korea	Parallel	PWV		Photoplethysmography	Chest press, leg press, shoulder press, abdominal crunches, leg curls, leg extensions, low back extensions, biceps curls and triceps extension followed by 20 minute of treadmill walking
Ho et al. 2012 [5]	Australia	Parallel	PWA	67-74%	SphygmaCor/AT	Leg press, leg curl, leg extension, bench press, rear deltoid row and treadmill
Kawasaki et al. 2011 [16]	Japan	Parallel	PWV	92%	Photoplethysmography	Land-based muscle strength groups, swimming and cycling for 2 hours
Loimaala et al. 2009 [17]	Finland	Parallel	PWV	75%	Photoplethysmography	Twice weekly aerobic training (jogging or walking) and twice weekly resistance training of large muscle groups
Miura et al. 2008 1DW [18]	Japan	Parallel	PWV		BP-203RPEII	Rubber tubes and/or lightweight dumbbell and walking or running
Miura et al. 2008 2DW [18]	Japan	Parallel	PWV		BP-203RPEII	Rubber tubes and/or lightweight dumbbell and walking or running
Ohta et al. 2012 [19]	Japan	Parallel	PWV	85%	Photoplethysmography	Bench step exercise
Okamoto et al. 2007 BRT [20]	Japan	Parallel	PWV		Form PWV/ABI	Running (aerobic) and resistance (chest and shoulder press, arm curls, seated rows, leg curls, leg press and sit-up)
OKamoto et al. 2007 ART [20]	Japan	Parallel	PWV		Form PWV/ABI	Running (aerobic) and resistance (chest and shoulder press, arm curls, seated rows, leg curls, leg press and sit-up)
Stewart et al. 2005 [21]	USA	Parallel	PWV	88%		Aerobic (treadmill, cycling, stairstepper) and resistance (latissmus dorsi pull down, leg extension, leg press, leg curl, bench press, shoulder press, seated row)
Wong et al. 2014 [22]	USA	Parallel	PWV, PWA		AT	Stretching exercise of major muscle groups

References

1. Beck DT, Martin JS, Casey DP, Braith RW: Exercise training reduces peripheral arterial stiffness and myocardial oxygen demand in young prehypertensive subjects. American Journal of Hypertension 2013, 26(9):1093-1102.
2. Cortez-Cooper MY, Anton MM, Devan AE, Neidre DB, Cook JN, Tanaka H: The effects of strength training on central arterial compliance in middle-aged and older adults. European Journal of Cardiovascular Prevention & Rehabilitation 2008, 15(2):149-155.
3. Croymans DM, Krell SL, Oh CS, Katiraie M, Lam CY, Harris RA, Roberts CK: Effects of resistance training on central blood pressure in obese young men. J Hum Hypertens 2014, 28(3):157-164.
4. Heffernan KS, Yoon ES, Sharman JE, Davies JE, Shih YT, Chen CH, Fernhall B, Jae SY: Resistance exercise training reduces arterial reservoir pressure in older adults with prehypertension and hypertension. Hypertension Research - Clinical & Experimental 2013, 36(5):422-427.
5. Ho SS, Radavelli-Bagatini S, Dhaliwal SS, Hills AP, Pal S: Resistance, aerobic, and combination training on vascular function in overweight and obese adults. Journal of Clinical Hypertension 2012, 14(12):848-854.
6. Miyachi M, Kawano H, Sugawara J, Takahashi K, Hayashi K, Yamazaki K, Tabata I, Tanaka H: Unfavorable effects of resistance training on central arterial compliance: a randomized intervention study. Circulation 2004, 110(18):2858-2863.
7. Okamoto T, Masuhara M, Ikuta K: Effects of eccentric and concentric resistance training on arterial stiffness. Journal of Human Hypertension 2006, 20(5):348-354.
8. Okamoto T, Masuhara M, Ikuta K: Effects of low-intensity resistance training with slow lifting and lowering on vascular function. J Hum Hypertens 2008, 22(7):509-511.
9. Okamoto T, Masuhara M, Ikuta K: Upper but not lower limb resistance training increases arterial stiffness in humans. European Journal of Applied Physiology 2009, 107(2):127-134.
10. Okamoto T, Masuhara M, Ikuta K: Effects of muscle contraction timing during resistance training on vascular function. Journal of Human Hypertension 2009, 23(7):470-478.
11. Okamoto T, Masuhara M, Ikuta K: Effect of low-intensity resistance training on arterial function. European Journal of Applied Physiology 2011, 111(5):743-748.
12. Okamoto T, Masuhara M, Ikuta K: Low-intensity resistance training after high-intensity resistance training can prevent the increase of central arterial stiffness. International Journal of Sports Medicine 2013, 34(5):385-390.
13. Yoshizawa M, Maeda S, Miyaki A, Misono M, Saito Y, Tanabe K, Kuno S, Ajisaka R: Effect of 12 weeks of moderate-intensity resistance training on arterial stiffness: a randomised controlled trial in women aged 32-59 years. British Journal of Sports Medicine 2009, 43(8):615-618.
14. Dobrosielski DA, Gibbs BB, Ouyang P, Bonekamp S, Clark JM, Wang NY, Silber HA, Shapiro EP, Stewart KJ: Effect of exercise on blood pressure in type 2 diabetes: a randomized controlled trial. Journal of General Internal Medicine 2012, 27(11):1453-1459.
15. Figueroa A, Park SY, Seo DY, Sanchez-Gonzalez MA, Baek YH: Combined resistance and endurance exercise training improves arterial stiffness, blood pressure, and muscle strength in postmenopausal women. Menopause 2011, 18(9):980-984.
16. Kawasaki T, Sullivan CV, Ozoe N, Higaki H, Kawasaki J: A long-term, comprehensive exercise program that incorporates a variety of physical activities improved the blood pressure, lipid and glucose metabolism, arterial stiffness, and balance of middle-aged and elderly Japanese. Hypertension Research - Clinical & Experimental 2011, 34(9):1059-1066.
17. Loimaala A, Groundstroem K, Rinne M, Nenonen A, Huhtala H, Parkkari J, Vuori I: Effect of long-term endurance and strength training on metabolic control and arterial elasticity in patients with type 2 diabetes mellitus. American Journal of Cardiology 2009, 103(7):972-977.

18. Miura H, Nakagawa E, Takahashi Y: Influence of group training frequency on arterial stiffness in elderly women. European Journal of Applied Physiology 2008, 104(6):1039-1044.
19. Ohta M, Hirao N, Mori Y, Takigami C, Eguchi M, Tanaka H, Ikeda M, Yamato H: Effects of bench step exercise on arterial stiffness in post-menopausal women: contribution of IGF-1 bioactivity and nitric oxide production. Growth Hormone & Igf Research 2012, 22(1):36-41.
20. Okamoto T, Masuhara M, Ikuta K: Combined aerobic and resistance training and vascular function: effect of aerobic exercise before and after resistance training. Journal of Applied Physiology 2007, 103(5):1655-1661.
21. Stewart KJ, Bacher AC, Turner KL, Fleg JL, Hees PS, Shapiro EP, Tayback M, Ouyang P: Effect of exercise on blood pressure in older persons: A randomized controlled trial. Archives of Internal Medicine 2005, 165(7):756-762.
22. Wong A, Figueroa A: Eight weeks of stretching training reduces aortic wave reflection magnitude and blood pressure in obese postmenopausal women. Journal of Human Hypertension 2014, 28(4):246-250.
