# Supplementary material for: Effects of Exercise Modalities on Arterial Stiffness and Wave Reflection: A Systematic Review and Meta-Analysis of Randomized Controlled Trials
Source: PLoS One. 2014 Oct 15;9(10):e110034. doi: 10.1371/journal.pone.0110034 (PMC4198209; doi:10.1371/journal.pone.0110034)
Supplement: Table S3 — Subgroup analyses of the effects aerobic exercise on augmentation index using a random-effects model. (PDF) [file pone.0110034.s007.pdf]

Table S3: Subgroup analyses of the effects aerobic exercise on augmentation index using a random-effects model

	Trials* (No.)	AIx (%)	95% CI	*I* (%)	*P Value***
Age of participants (years)					**0.18**
≤ 50 years	7 (215)	-4.01	-6.82 to -1.18	61.6	
> 50 years	9 (223)	-0.98	-5.47 to 3.50	86.5	
Body mass index (kg/m^2)					**0.86**
≤ 24.9	3 (76)	-3.86	-11.17 to 3.43	61.6	
≥ 25	13 (362)	-2.46	-5.56 to 0.63	83.6	
Baseline systolic pressure (mmHg)					**0.41**
≤ 120	8 (228)	-2.09	-5.68 to 1.50	86.6	
> 120	8 (210)	-4.99	-6.54 to -3.45	0	
Baseline diastolic pressure (mmHg)					**0.81**
≤ 80	13 (354)	-2.56	-5.43 to 0.30	84.6	
> 80	3 (84)	-3.65	-8.80 to 1.49	0	
Baseline augmentation index (%)					**0.99**
≤ 11	7 (203)	-2.68	-6.52 to 1.15	88.5	
> 11	8 (202)	-3.49	-5.91 to -1.08	14.3	
Intensity of exercise (METs)					**0.47**
≤ 7	7 (186)	-1.88	(-7.26 to -3.50)	64.5	
> 7	8 (234)	-2.72	(-5.12 to -0.31)	53	
Duration of the study (weeks)					**0.38**
≤ 10	4 (101)	-2.80	-10.49 to 4.88	78	
> 10	12 (337)	-3.15	-5.14 to -1.15	53	
Quality of studies					**0.21**
< 3	7 (188)	-1.48	-5.26 to 2.29	75.2	
≥ 3	9 (250)	-4.79	-6.01 to -3.59	0	

*Number of trials or subgroups (number of participants)

***P* value for the meta-regression analyses between subgroups. WMD: weighted mean difference
